# Personalization of human body models and beyond via image registration

**DOI:** 10.3389/fbioe.2023.1169365

**Published:** 2023-05-19

**Authors:** Xiaogai Li, Qiantailang Yuan, Natalia Lindgren, Qi Huang, Madelen Fahlstedt, Jonas Östh, Bengt Pipkorn, Lotta Jakobsson, Svein Kleiven

**Affiliations:** ^1^ Division of Neuronic Engineering, Department of Biomedical Engineering and Health Systems, KTH Royal Institute of Technology, Huddinge, Sweden; ^2^ Mips AB, Täby, Sweden; ^3^ Volvo Cars Safety Centre, Gothenburg, Sweden; ^4^ Division of Vehicle Safety, Department of Mechanics and Maritime Sciences, Chalmers University of Technology, Gothenburg, Sweden; ^5^ Autoliv Research, Vargarda, Sweden

**Keywords:** finite element human body model, image registration, mesh morphing, personalized simulations, traffic safety

## Abstract

Finite element human body models (HBMs) are becoming increasingly important numerical tools for traffic safety. Developing a validated and reliable HBM from the start requires integrated efforts and continues to be a challenging task. Mesh morphing is an efficient technique to generate personalized HBMs accounting for individual anatomy once a baseline model has been developed. This study presents a new image registration–based mesh morphing method to generate personalized HBMs. The method is demonstrated by morphing four baseline HBMs (SAFER, THUMS, and VIVA+ in both seated and standing postures) into ten subjects with varying heights, body mass indices (BMIs), and sex. The resulting personalized HBMs show comparable element quality to the baseline models. This method enables the comparison of HBMs by morphing them into the same subject, eliminating geometric differences. The method also shows superior geometry correction capabilities, which facilitates converting a seated HBM to a standing one, combined with additional positioning tools. Furthermore, this method can be extended to personalize other models, and the feasibility of morphing vehicle models has been illustrated. In conclusion, this new image registration–based mesh morphing method allows rapid and robust personalization of HBMs, facilitating personalized simulations.

## 1 Introduction

Finite element (FE) human body models (HBMs) are becoming increasingly important numerical tools in vehicle safety for understanding injury mechanisms and developing prevention strategies (Östh et al., 2015; Boyle et al., 2019; Hu et al., 2019; Jakobsson et al., 2019; Pipkorn et al., 2019; Boyle et al., 2020; Hwang et al., 2020; Tang et al., 2020; Grebonval et al., 2021; Leledakis et al., 2021; Larsson et al., 2022; Bohman et al., 2022; Booth et al., 2022; Corrales et al., 2022; Erlinger et al., 2022; Mishra et al., 2022; Piqueras et al., 2022; Putra et al., 2022; Östh et al., 2022). HBMs have advantages over crash test dummies, such as representing varying loading directions, different anthropometries and sex, and muscle tonus (Jakobsson et al., 2019). Examples of HBMs include the THUMS (Shigeta et al., 2009), GHBMC (Gayzik et al., 2012), VIVA+ (John et al., 2022), SAFER HBM (Pipkorn et al., 2021), and PIPER (Beillas et al., 2016).

Personalized HBMs are required in situations where the individual anatomy is important, such as in the reconstruction of post-mortem human subject (PMHS) impact for HBM validation or accident reconstructions to elucidate injury mechanisms. However, developing a validated and reliable HBM from the start is time-consuming and remains challenging. Mesh morphing is a more efficient alternative for generating personalized HBMs than generating one from the start, which involves moving the nodes of the baseline HBM into a subject by using a displacement field that reflects the anatomical differences between the two. A literature review by Hu et al. (2012) highlighted the capacity of thin-plate spline radial basis function (RBF) for personalizing HBMs to a diverse population. Vavalle et al. (2014) morphed a GHBMC 50th-percentile male model into a 95th-percentile male, while Schoell et al. (2015) morphed the same into a 65-year-old (65YO) male. A study by Jolivet et al. (2015) also demonstrated that mesh morphing can generate HBMs with reasonable mesh quality and geometry accuracy by properly defining geometry targets; Beillas and Berthet (2017) morphed the GHBMC of a 95th male and a 5th female to 52 subjects based on anthropometric data. A series of studies from the University of Michigan have developed methodologies for generating parametric HBMs for a diverse population based on statistical shape models (SSMs) (Shi et al., 2015; Hwang et al., 2016a; Hwang, Hallman et al., 2016b; Hu et al., 2016; Zhang et al., 2017; Hu et al., 2019; Hwang et al., 2020; Tang et al., 2020), and this method has been adopted by John et al. (2022) to morph a 50th-percentile VIVA+ female model from seated to standing postures and a 50th male.

In addition to the abovementioned work, the open-source PIPER software has been developed in a European PIPER project, enabling personalization and positioning of HBMs through kriging (Jolivet et al., 2015; Janak et al., 2018; Janak et al., 2021), an interpolation method to deform geometrical models based on a set of sources and the associated target control points. The software is accompanied by a 6YO child model that is scalable from a 1.5YO to 12YO through metadata files containing landmarks and control points. The PIPER software has successfully been used to personalize and position various HBMs such as GHBMC (Grebonval et al., 2021; Corrales et al., 2022; Erlinger et al., 2022), THUMS (Germanetti et al., 2020), and VIVA (Kleinbach et al., 2018), demonstrating the versatility of the PIPER software beyond positioning the paired PIPER child model (Giordano et al., 2017; Li and Kleiven, 2018).

The abovementioned studies applied RBF or kriging interpolation methods to personalize the HBMs, which according to Janak et al. (2021), leads to an identical interpolation function for the parameters used in these studies. Both RBF and kriging require the identification of landmarks on the skin and skeleton/joints of both the baseline HBM (as *source*) and the subject (as *target*), and the landmarks have to correspond between the two. Based on these landmarks, a displacement field is calculated to move the baseline HBM nodes into the *target* subject. However, identifying the landmarks can be time-consuming and requires manual effort, which has led to the development of methods to simplify the process (Wu et al., 2019). The PIPER software uses a kriging method and requires metadata files with landmarks to personalize and position HBMs (PIPER Software Framework and Application: User guide, 2017), and new metadata have to be developed for new HBMs.

Both the RBF and kriging morphing methods are computationally expensive due to the task of inverting a large matrix. The RBF can handle a few thousand landmarks that define body posture and external surface shape, but for more accurate results with hundreds of thousands of landmarks, it becomes computationally challenging. To address this issue, a regional mesh morphing strategy has been proposed to split the body into parts and perform the morphing sequentially (Zhang et al., 2017; John et al., 2022). Additionally, a workflow has been suggested to efficiently handle the computation by using iterative subsampling and spatial subdivision methods (Janak et al., 2021).

Our study introduces a new method to personalize HBMs using image registration. We aim to overcome previous challenges associated with landmark-based morphing and offer a rapid and effective way to personalize HBMs. Recent studies have shown that image registration–based mesh morphing can be effective for personalizing human brain models (Giudice et al., 2020; Li, 2021; Li et al., 2021). However, it remains to be decided whether this method can be extended to personalize whole-body HBMs, in particular with the inclusion of skeletons. Further investigation is required to explore the feasibility and potential benefits of using image registration–based mesh morphing for whole-body HBMs, which motivates this study. We also explored the application of the method for geometrical correction and evaluated its potential in personalizing vehicle models.

## 2 Methods

Section 2.1 describes an overview of how we applied the image registration–based morphing method to four baseline HBMs and morphed them into ten subjects to demonstrate its effectiveness. The details of the method are explained in Section 2.2, followed by a demonstration of the usage of the method in Section 2.3. Finally, evaluations of morphed models are presented, which include element quality and runnability tests (Section 2.4).

### 2.1 HBM personalization: baseline HBMs and subjects

**FIGURE 1:**
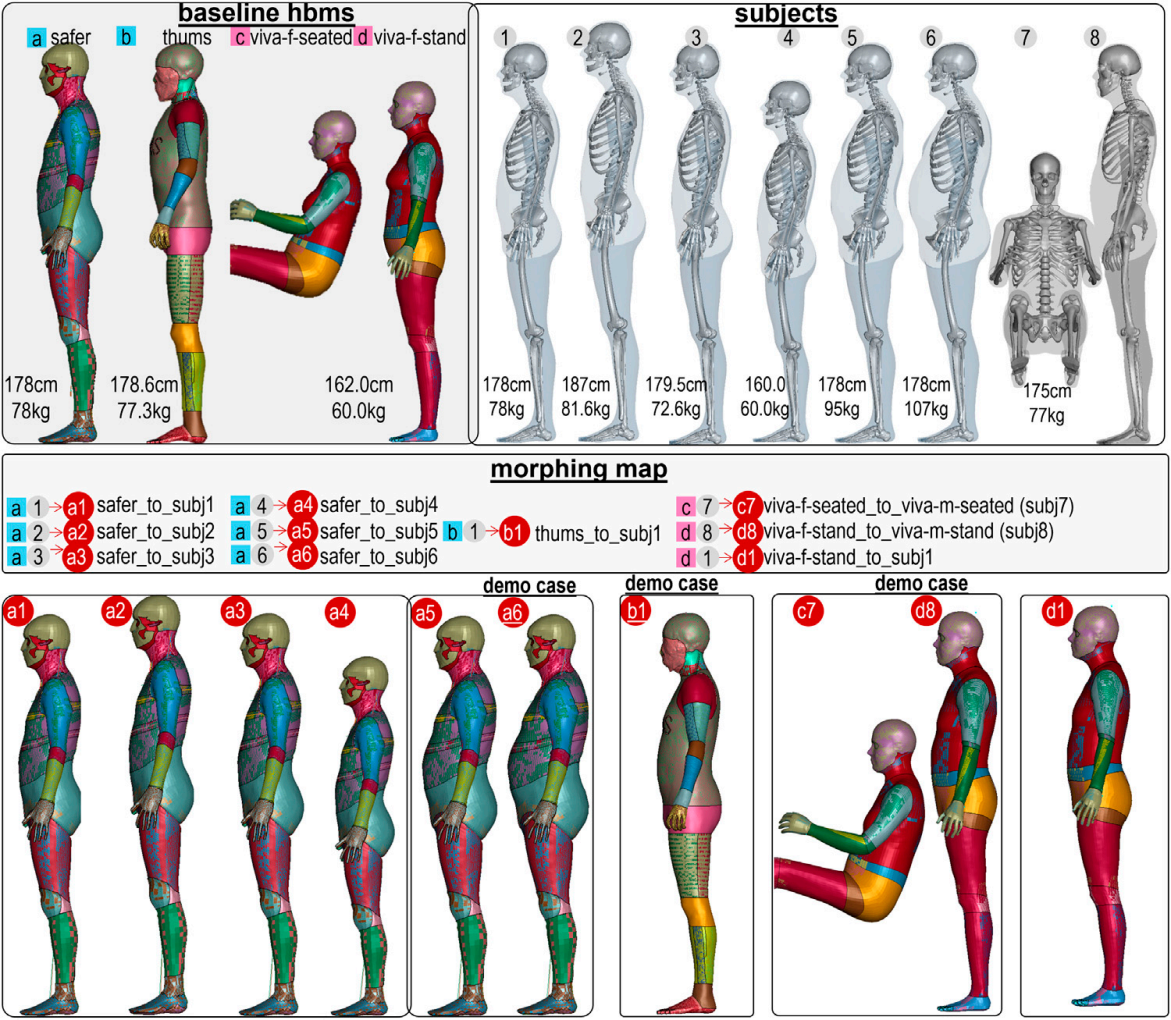
HBM personalization morphing map. Four baseline HBMs were morphed into eight subjects with varying heights (ranging from 160 to 187 cm) and a BMI of up to 34. SAFER HBM was morphed into six subjects (*subj1* to *subj6*), THUMS was morphed into one subject (*subj1*), and the VIVA+ female was morphed into its corresponding male for both seated (*subj7*) and standing (*subj8*) postures. Additionally, the VIVA+ female standing model was morphed into *subj1*.

The four baseline HBMs include two models of 50th-percentile males in the standing posture (SAFER HBM and THUMS V4.02) and two models of 50th-percentile females in the seated and standing postures (VIVA+) (illustrated in Figure 1 and listed in Table 1). The anthropometric data of the eight target subjects are in Table 2. In total, ten personalized models were generated, and some were morphed into the same subject (*subj1*) as shown in the morphing map (Figure 1 and Table 3). The subsequent sections provide further information on the baseline HBMs and body shapes of the target subjects.

**TABLE 1:** Anthropometric data of the four baseline HBMs.

Baseline HBM	Standing height (cm)	Weight (kg)	Reference
SAFER HBM (male)	178.0	78.0	Lindgren et al. (2023)
THUMS (male)	178.6	77.3	Shigeta et al. (2009)
VIVA+ seated (female)	162.0	60.0	John et al. (2022)
VIVA+ standing (female)			

**TABLE 2:** Anthropometric data for the eight subjects for personalizing baseline HBMs.

Subject no.	Standing height (cm)	Weight (kg)	Sex	Note
subj1	178.0	78.0	male	PMHS subjects from the study Forman et al. (2015)
subj2	187.0	81.6	male	
subj3	179.5	72.6	male	
subj4	160.0	60.0	male	A real-world accident database
subj5	178.0	95.0	male	BMI = 30
subj6	178.0	107.0	male	BMI = 34
subj7	175.0	77.0	male	Average male John et al. (2022)
subj8				

Note: Since the current study only used anthropometric values to generate body shape models, written informed consent was not required.

**TABLE 3:** List of the ten personalized HBMs from four baseline.

a1: SAFER 50 male personalized to subj1 (50th 178 cm, 78 kg)
a2: SAFER 50 male personalized to subj2 (187 cm)
a3: SAFER 50 male personalized to subj3 (179 cm)
a4: SAFER 50 male personalized to subj4/160 cm)
a5: SAFER 50 male personalized to subj5 (BMI = 30)
a6: SAFER 50 male personalized to subj6 (BMI = 34)
b1: THUMS 50th male personalized to 50th male subj1 (subj1)
c7: VIVA+ 50th female seated personalized to VIVA+ 50th male seated (subj7)
d8: VIVA+ 50th female standing personalized to VIVA+ 50th male standing (subj8)
d1: VIVA+ 50th female standing personalized to subj1
b1’: THUMS 50th male personalized to 50th male subj1 using the “shielding” pipeline

#### 2.1.1 Baseline HBMs

##### 2.1.1.1 SAFER-pedestrian HBM

A pedestrian version of the SAFER HBM was used, which was developed by Lindgren et al. (2023), through positioning and morphing of the SAFER HBM v10 in a seated position (Pipkorn et al., 2021). The following steps were taken: first, the SAFER seated occupant was positioned to a standing posture using the software Oasys PRIMER (Oasys, Solihull, United Kingdom). This led to a standing postured model but with distorted buttocks and a shorter standing body height. This PRIMER-positioned model was then geometrically corrected by morphing it to a 50th-percentile male body shape (see Supplementary Appendix S1), leading to the baseline pedestrian SAFER HBM. This model was then personalized to target subjects. Further details of the development and validation of the pedestrian SAFER HBM are presented in the study by Lindgren et al. (2023).

##### 2.1.1.2 THUMS 50th pedestrian male

THUMS V4.02 pedestrian model was downloaded from the free access data set (https://www.toyota.co.jp/thums/download). The original model’s arm was rotated slightly within the software Oasys PRIMER to create the baseline model, ensuring that the arms were in a similar position to that of the target subjects for further personalization in this study.

##### 2.1.1.3 VIVA+ HBMs in seated and standing postures

The 50th-percentile females in seated and standing postures (labeled as *50F-seated* and *50F-standing*) were downloaded from an open-source data set (https://openvt.eu/fem/viva/vivaplus/-/tree/main/model) and used as the baseline models to generate personalized HBMs for their corresponding male subjects. Note that the male versions of the VIVA+ model (labeled as *50M-seated* and *50M-stand*) were also downloaded. These models were previously morphed from their female counterparts using an RBF method (John et al., 2022). The inclusion of the male versions of the HBMs served two purposes: 1) to reverse engineer the body shapes to which the female models were morphed and 2) to compare the morphed models with the current method in this study with the RBF approach (Section 3.5).

#### 2.1.2 Body and skeleton shapes of target subjects

For subjects 1–6, the body shapes of the skin and skeleton were generated based on previously developed statistical skin and skeleton models of SMPL (Loper et al., 2015), SMPLX (Bogo et al., 2016), and OSSO (Keller et al., 2022). Briefly, the subject’s anthropometric information, such as height, weight, and sex, was used as input to generate a SMPLX skin surface of the subject. The SMPLX was then positioned to match the stance of the baseline HBM using the SMPLX built-in tools. The surface model was then converted to SMPL format to allow subsequent generation of the corresponding skeleton of the positioned skin using the OSSO algorithm (OSSO: Obtaining Skeletal Shape from Outside) presented by Keller et al. (2022).

For subjects 7 and 8, the skin and skeletons were reverse-engineered from the VIVA+ male models of the seated and standing positions, respectively, by extracting the skin and skeleton surfaces from the downloaded *50M-seated* and *50M-stand* HBMs.

### 2.2 Method for HBM personalization via image registration

**FIGURE 2:**
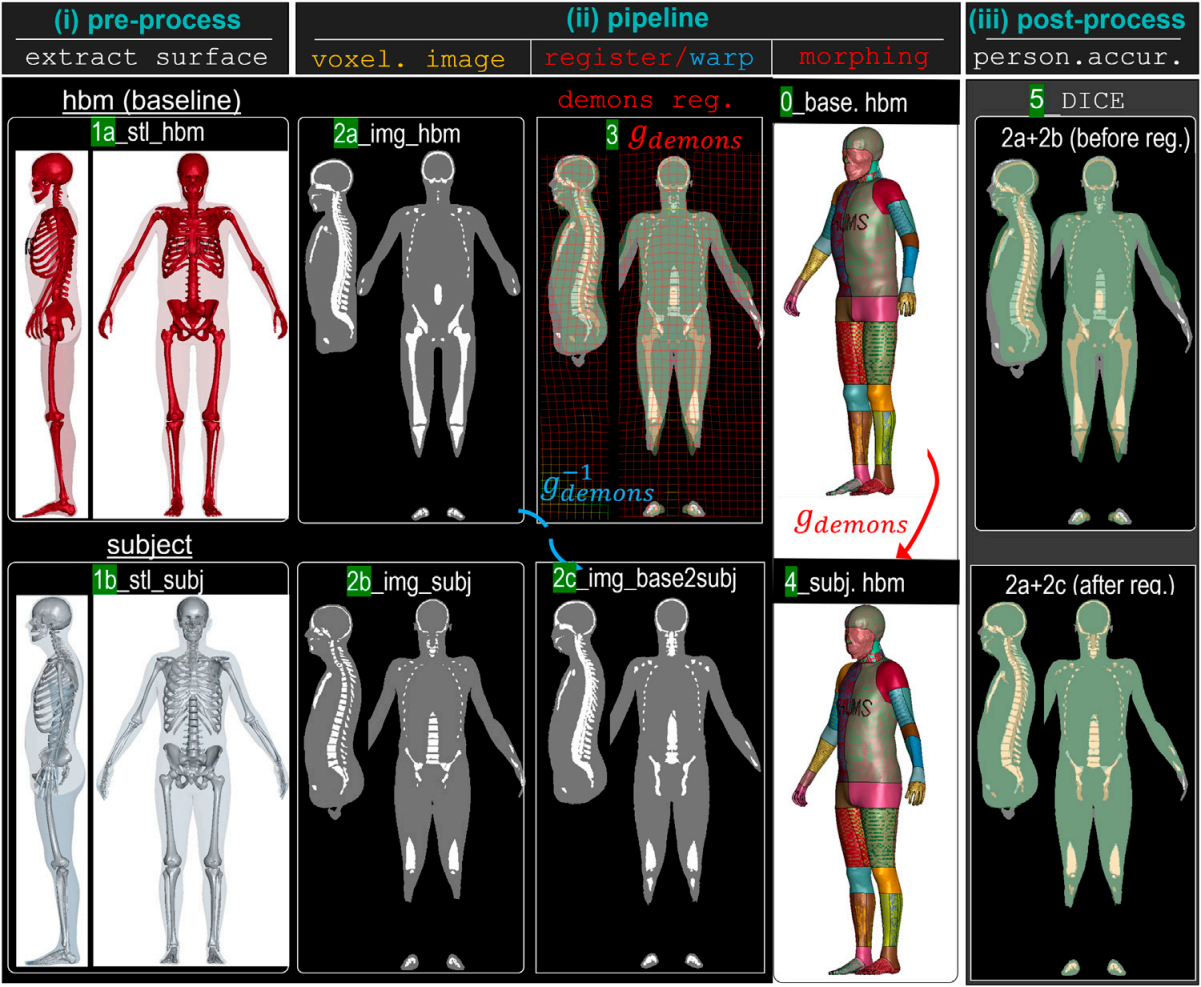
Overview of the image registration–based mesh morphing method for HBM personalization, which included three modules: (i) pre-processing extracts body shapes from the baseline HBM (*1a_stl_hbm*) and subject (*1b_stl_subj*) and converts them into binary images (*2a_img_hbm* and *2b_img_subj*); (ii) Demons registration is performed with *2a_img_hbm* as the *fixed* image and *2b_img_subj* as the *moving* image to obtain a displacement field ( $g_{demons}$ ). This displacement field, defined on the *fixed* image space, is used to morph the nodes of the baseline HBM (*0_baseline_hbm*) into a personalized HBM (*4_subject_hbm*) that corresponds to the target subject. The image corresponding to the baseline HBM (*2a_img_hbm*) is then warped by the inverse of the displacement field ( $g_{demons}^{-1}$ ), resulting in the warped image *2c_img_basewarped2subj*, which corresponds to the personalized HBM (*4_subject_hbm*). Comparing image *2c_img_basewarped2subj* with the golden truth image *2a_img_subj* allows for quantifying personalization accuracy, as measured by *5_DICE.*

The image registration–based morphing method consists of three modules: i) pre-processing, ii) image registration pipeline, and iii) post-processing. Pre-processing involves converting the shape of the human body, that includes the skin and skeleton, into binary images through voxelization. The pipeline uses Demons registration to obtain a displacement field that represents the anatomical differences between the baseline HBM and target subject, which is then used to morph the baseline into a personalized HBM. Post-processing assesses the accuracy of the personalization. Figure 2 illustrates the morphing method applied to personalize THUMS to subject 1. Further details on each module are supplied below.

#### 2.2.1 Pre-processing to voxelize body shape and skeleton to a binary image

The surface model of the baseline HBM and subject, which includes the skin surface and skeleton, are converted into binary images by 1) determining the minimum and maximum of the xyz coordinates of the polygon mesh, 2) defining the resolution of the output image, 3) voxelizing the inside as 1 and 2 and the outside as 0, and 4) outputting the binary image with flesh image value 1, skeleton 2, and background 0. The voxelization step is done for both the flesh and skeleton using the “Convert model to segmentation node” module in Slicer 3D, which allows the easy conversion of surface models into binary images.

#### 2.2.2 Demons image registration

In this study, a non-linear registration method, the Diffeomorphic Demons algorithm (Vercauteren et al., 2009) implemented in the open-source software 3D Slicer was used. This algorithm was chosen for its ability to handle large anatomical differences, as demonstrated in previous studies (von Holst et al., 2012; Li et al., 2013; von Holst and Li, 2013; Li, 2021; Li, Zhou, and Kleiven, 2021). The binary images of the baseline HBM and subject are first rigidly aligned, then Demons registration is performed, where the subject’s image serves as the *moving* image and the baseline image as the *fixed image*. The registration process calculates a displacement field that aligns the two images as accurately as possible. Note that a smooth factor of 2 is chosen for Demons registration which is shown to be efficient for all the tested cases and allows a smooth displacement field capturing the anatomical differences between the HBMs and subjects, while allowing to handle different internal organ shapes between the two.

#### 2.2.3 Mesh morphing

The displacement field $g_{demons}$ obtained from the abovementioned registration step is defined for every voxel in the *fixed* image. As the baseline HBM is in the same space as the *fixed* image, applying $g_{demons}$ to the baseline HBM leads to a personalized HBM. This is achieved by morphing the nodes of the baseline mesh to new positions using the formula:

$$x^{i}=X^{i}+u^{i},$$ (1)

where $X^{i}$ is the nodal coordinate of node i , $u^{i}$ is the linearly interpolated displacement vector at node n from $g_{demons}$ , and $x^{i}$ is the updated nodal coordinate. The personalized HBM is formed by the morphed nodes and same element definitions as the baseline.

#### 2.2.4 Post-process for personalization accuracy: DICE and HD95 distance

The registration accuracy is evaluated by calculating the DICE and 95th-percentile Hausdorff distance (HD95) between a warped image ( $img_{warped}$ ) and the subject’s image. The baseline image ( $img_{baseline}$ ) is warped via the inverse of displacement fields from each registration step ( $g_{demons}^{-1}$ ) resulting in a warped image ( $img_{warped}$ )

$$img_{warped}=g_{demons}^{-1}\left(img_{baseline}\right).$$ (2)

As $img_{warped}$ corresponds to the personalized model, therefore both metrics also reflect the personalization accuracy.

DICE is a single metric to measure the spatial overlap between images defined as twice the number of elements common to both sets divided by the sum of the number of elements in each set (Ou et al., 2014).

$$DICE\left(A,B\right)=\frac{2\left|A\cap B\right|}{\left|A\right|+\left|B\right|},$$ (3)

where A and B denote the binary segmentation labels, $\left|A\right|$ and $\left|B\right|$ are the number of voxels in each set, and $\left|A\cap B\right|$ is the number of shared voxels by A and B ; a DICE value of 0 implies no overlap, whereas a DICE coefficient of 1 indicates perfect overlap between the warped and target images.

The Hausdorff distance is defined as

$$HD\left(C,D\right)=\max\left(h\left(C,D\right),h\left(D,C\right)\right),$$ (4)

where C,D are the two sets of vertices from two segmented images:

$$h\left(C,D\right)=\begin{array}{c} \max\\ c\in C \end{array}\;\begin{array}{c} \max\\ d\in D \end{array}\left\|c-d\right\|.$$ (5)

The 95th-percentile Hausdorff distance (HD95) is used following earlier studies (Ou et al., 2011; Ou et al., 2014). HD95 ranges from 0 to above 0, where a lower value indicates better registration accuracy between the warped and target images. Note that when there is a substantial difference in the internal organ shape between the HBMs and subjects, a decreased registration accuracy is expected. This is because, to maintain valid element quality, Demons registration with a smooth factor of 2 is used to morph the baseline HBM to the subjects, accounting for the overall shape, which can result in lower accuracy.

### 2.3 Personalization pipelines and demonstration applications

Three typical personalization pipelines (types I, II, and III) are described, along with their applications in generating HBMs *a6*, *b1*, and *d8*.

Type I is a basic pipeline (illustrated in Figure 2) that works well for most cases and has been used to generate nine personalized models except *a4*. An example usage of Type I is demonstrated by personalizing the SAFER HBM to a subject with a high BMI of 34 (*a6*) in Section 2.3.1. A skin-only parametric pipeline was also performed to emphasize the importance of the skeleton during morphing.

Type II pipeline adds extra steps to Type I for aligning subjects with significant anatomical differences with the baseline HBM. These extra steps can be a global transformation, multiple Demons registrations, or multiple morph steps focusing on local regions. For example, Type II was used to generate *a4* (subject 4 with a height of 160 cm, morphed from a baseline HBM pedestrian SAFER with a height of 178 cm). In this case, a global transformation was used to elongate the subject image by a factor calculated as 178/160, which was then used as the input for the subsequent Demons registration.

Type III “shielding” pipeline is used to intentionally prevent morphing parts of the HBM. The application of Type III is demonstrated by 1) the THUMS model, where the head is “shielded” from morphing, as detailed in Section 2.3.3, and 2) the SAFER model, as detailed in Supplementary Appendix S1.

Both demonstrations highlight the ability of this method for geometrical correction. To further illustrate the geometrical correction capacity, another demonstration is shown for correcting the PIPER 18YO head model, as detailed in Supplementary Appendix S3. We also demonstrate the application of the method for morphing vehicles. As the focus of this study is on HBM morphing, all parts related to vehicle morphing are presented in Supplementary Appendix S2.

#### 2.3.1 Type I pipeline application: morphing HBM to high-BMI subject (*a6*) and skin-only

**FIGURE 3:**
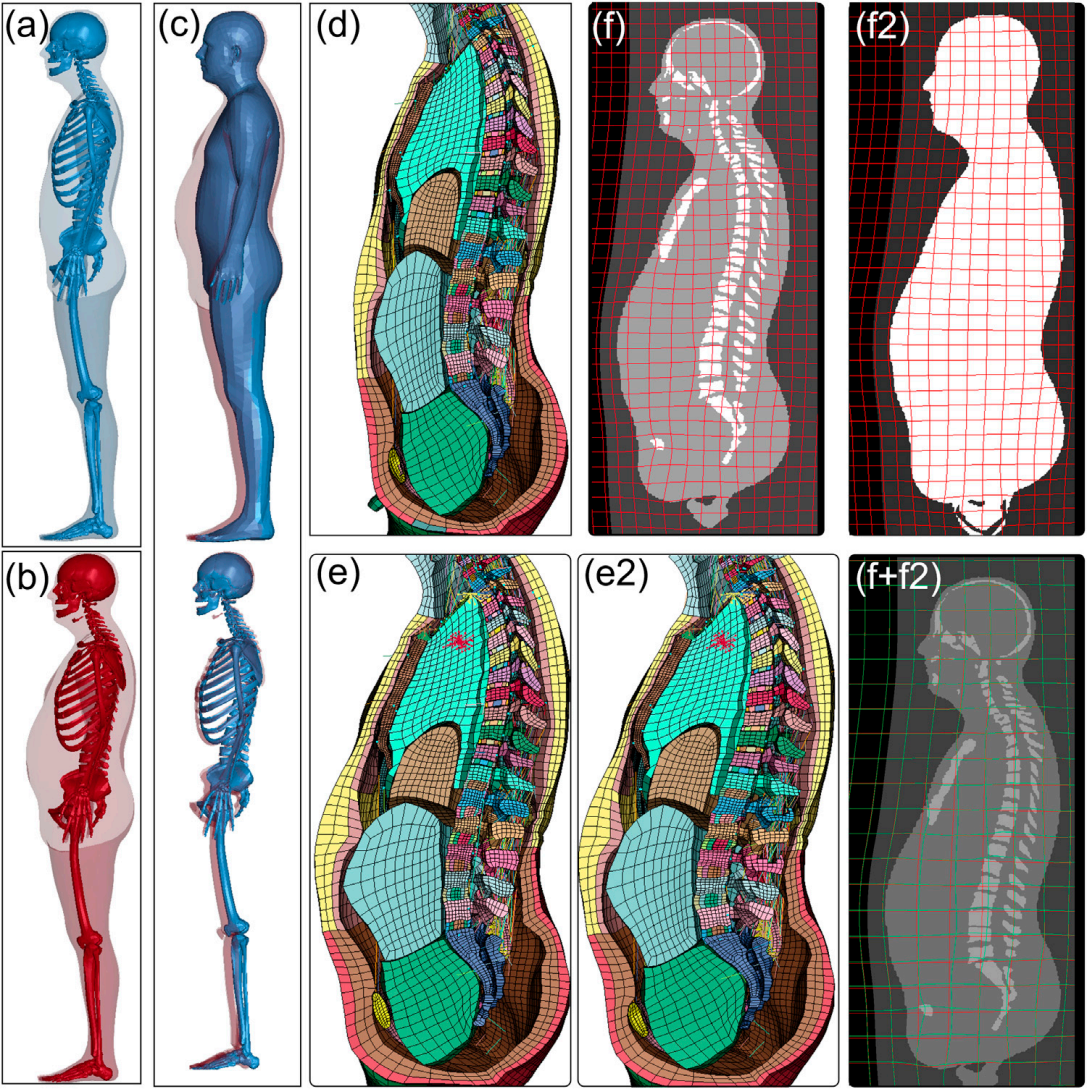
Baseline SAFER HBM **(A)** morphed to *subj6* with a BMI of 34 **(B)**, with an overlay that demonstrates that the skeleton differs less than the flesh of a person with high BMI than the baseline **(C)**. The mesh of the baseline **(D)** and personalized model **(E)** shows the expansion of the mesh at the belly which is deformed by the displacement field **(F)** obtained from the Demons registration with the skeleton included. A parametric pipeline is performed using body shape only without a skeleton, resulting in a morphed mesh **(E2)** by the displacement field **(F2)**. The comparison of the two displacement fields **(F** and **F2)** highlights the difference between the two (green frame shows baseline pipeline and red shows parametric pipeline).

Figure 3 displays the process of morphing the baseline SAFER HBM to subject 6 with a BMI of 34, along with the comparison of a parametric pipeline with skin only, while excluding the skeleton. The personalized mesh shows an expansion in the belly region when compared to the baseline HBM (Figure 3E vs. Figure 3D). Overlaying the baseline displacement field (in green frame lines, Figure 3F) with that obtained from the parametric pipeline (Figure 3F) highlights the difference between them and the need for including the skeleton in the registration step, especially for target subjects with high BMIs.

#### 2.3.2 Type I pipeline application: morphing female to male (*d8*)

**FIGURE 4:**
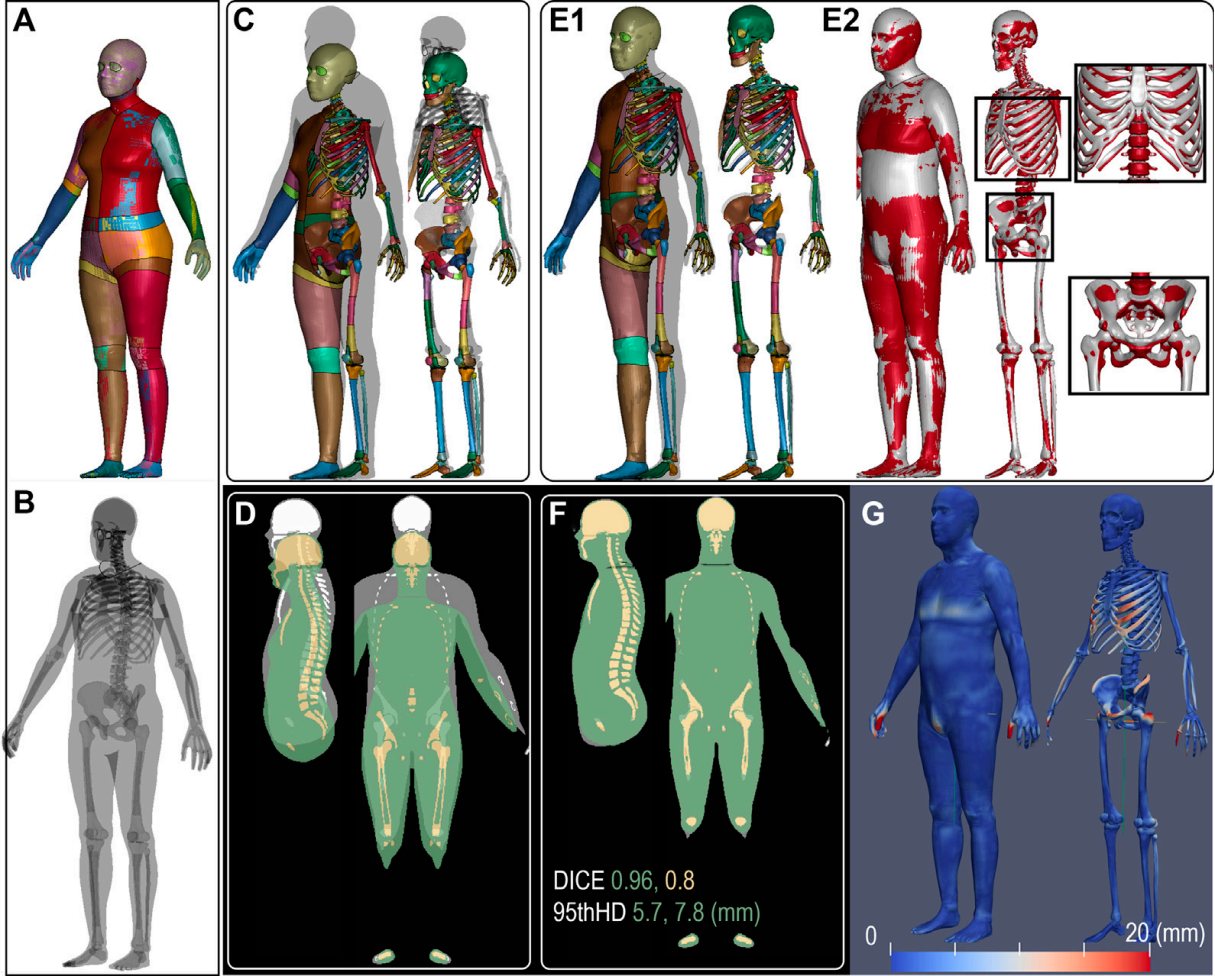
VIVA+ female in standing posture **(A)** was morphed to a subject (50th male) **(B)**, overlaying together shows the difference before morphing in shape **(C)** and the voxelized image **(D)** with the former in color and the latter in gray. The image after morphing **(F)** is used for calculating DICE for personalization accuracy, resulting in DICE 0.96 and HD95 5.7 mm. To depict personalization accuracy, the personalized male subject from the female baseline **(E1)** is superimposed onto the baseline female **(E2)**. And the absolute error of the nearest distance between the two surfaces was calculated and visualized for both the skin and skeleton surfaces **(G)**.

The VIVA+ female in a standing posture was morphed into a 50th male using Type I pipeline (as shown in Figure 4). The differences between the two before morphing are illustrated in Figure 4C and D, which become minimal after morphing (Figures 4E, F), resulting in a DICE score of 0.96 and HD95 of 5.7 mm for the flesh. The absolute distance errors for both the skin and skeleton are visualized in Figure 4G, showing the skin to be almost perfectly aligned except for the fingers with a larger difference. The average distance error for the skeleton is 2.39 mm (Figure 4G), with the largest error observed at the ribs and hip edge. Registration accuracy in terms of DICE and HD95 for all other subjects is presented in the Results section.

#### 2.3.3 Type III pipeline application: “shielding” and geometrical correction (*b1*)

**FIGURE 5:**
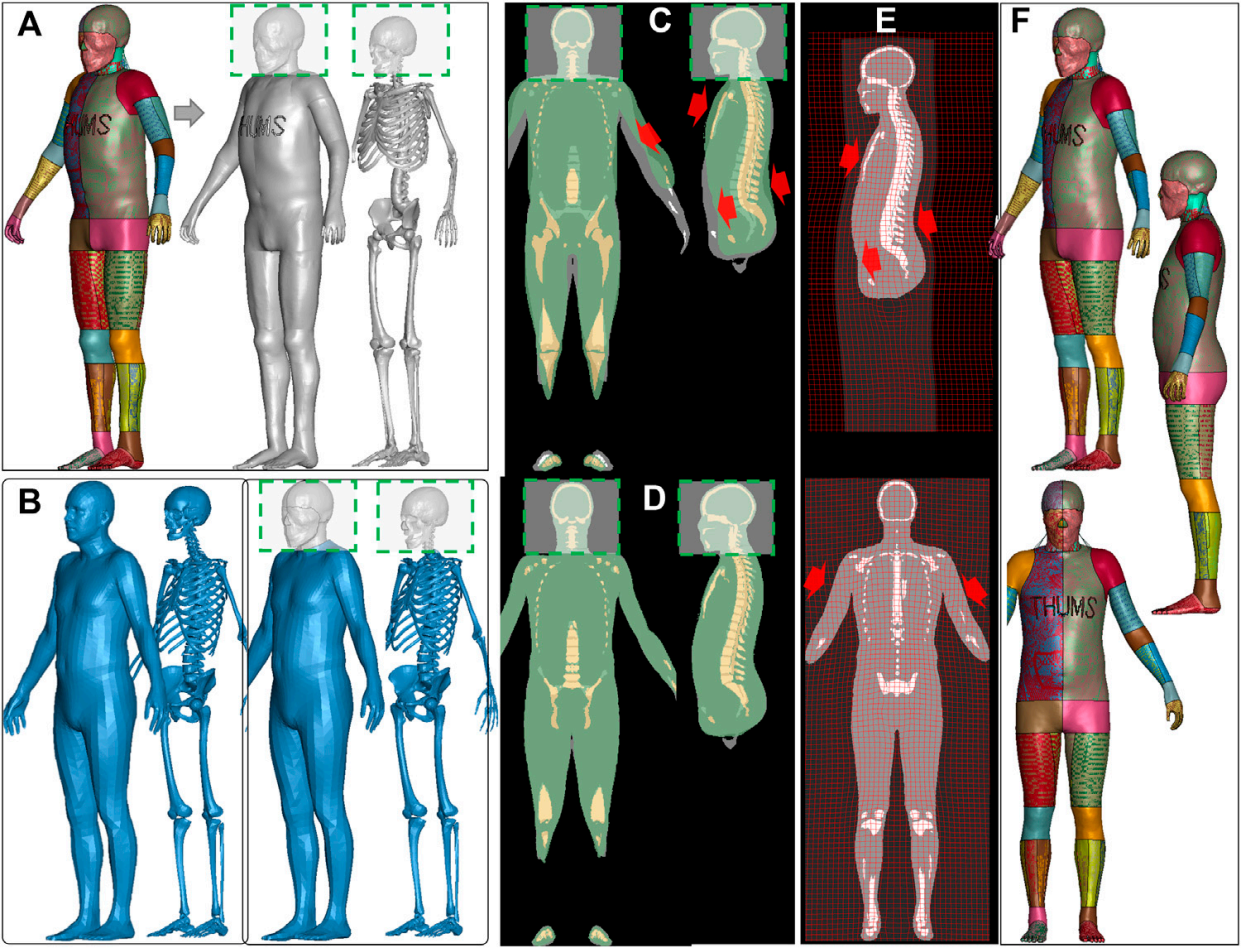
Baseline THUMS body and skeleton surfaces extracted **(A)** and installed to the head (scalp and skull) surfaces of the subject **(B)** to “shield” the head during morphing to geometrically morph other body part only with the head unmodified. Before morphing, a prominent chest is seen **(C)**, and after personalization, the difference between the subject (in gray) and the personalized image (in color) is almost invisible **(D)**. The displacement field shown in frame lines **(E)** illustrates the deformation of the chest, and the arm is slightly repositioned. **(F)** shows the final personalized HBM from THUMS using the Type III pipeline, which has a similar body to when using Type I pipeline (Figure 2, 4), but with an unmodified head.

The THUMS HBM has an unusually prominent chest, which is shown to be corrected through the current morphing method. The basic Type I pipeline used for morphing THUMS to *subj1* resulted in an unusual head shape (Figure 2 *4_subject_hbm*) due to the topological difference between the baseline and subject. To resolve this, the Type III pipeline was used to “shield” the difference in the head, meaning the head was not to be modified during morphing. This involved an additional step of replacing the head surface (scalp and skull) of the subject (Figure 5B) with that of THUMS (Figure 5A). Demons registration was then performed between the baseline HBM and subject (Figure 5C), which resulted in minimal displacement in the head region as indicated by the regular and undistorted frame lines (Figure 5E). The Type III pipeline led to a final personalized model from THUMS to subject 1(*b'*) with a corrected body and the prominent chest pushed down (Figure 5F). The arm was also slightly repositioned during the morphing as indicated by the red arrows (Figures 5C, E).

### 2.4 Analysis of personalized models: element quality and runnability test

Element quality was analyzed in the personalized HBMs in the form of *Jacobian* and *aspect ratio*, two commonly used indices for assessing element quality as reviewed earlier by Burkhart et al. (2013). Additionally, the minimum length of the elements was evaluated, as it determines the critical time step in the explicit FE dynamics analysis.

As HBMs contain complex contacts, a realizable morphing method should also ensure the maintaining of contacts, which are checked. The runnability of the personalized models was tested by subjecting them to a pedestrian impact to the side by a generic vehicle model developed by Pipkorn et al. (2014) at a velocity of 40 km/h. For details of the HBMs regarding materials, and interaction between organs, the readers are referred to the original study presenting these HBMs as mentioned in Section 2.1.1. The details of the generic vehicle model are found in the study by Pipkorn et al. (2014). LS-Dyna single precision version 13.0 was used to simulate the contact between the HBM and vehicle with a coefficient of 0.2.

## 3 Results

Personalization accuracy is presented in Section 3.1, followed by an analysis of element quality in Section 3.2 and runnability test results in Section 3.3. The analysis of all three HBMs morphed to the same subject can be found in Section 3.4, and a comparison between the current image registration–based approach and RBF is presented in Section 3.5.

### 3.1 Personalized HBMs and accuracy

**FIGURE 6:**
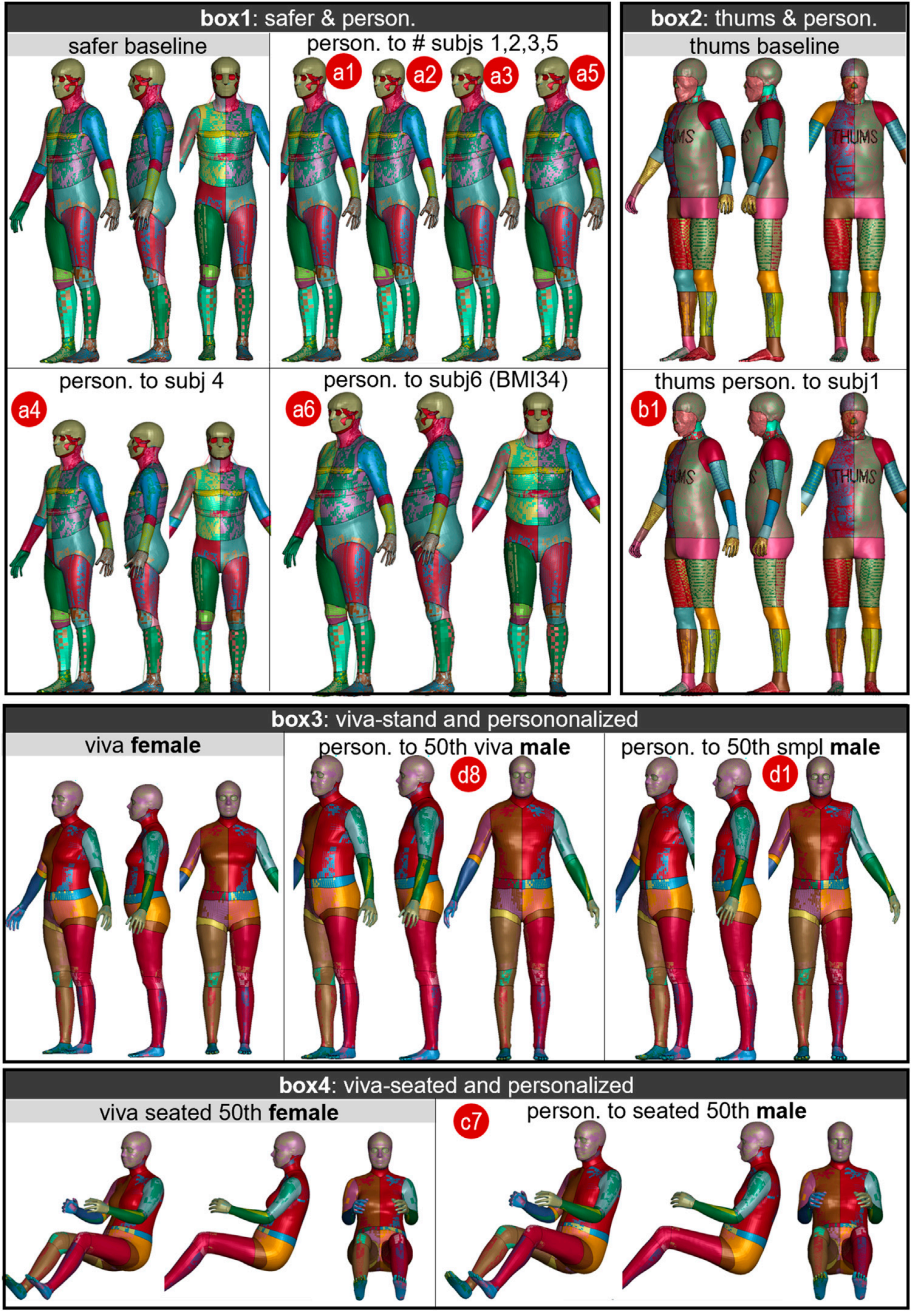
Ten personalized models morphed from four baseline HBMs. Each box shows the baseline HBM and its personalized models. Box1 shows SAFER HBM morphed to six subjects (*subj1* to *subj6*). Box2 shows THUMS morphed to subject 1. Box3 and Box4 show the VIVA+ female 50th morphed into a male version in standing and seated postures, respectively.

**FIGURE 7:**
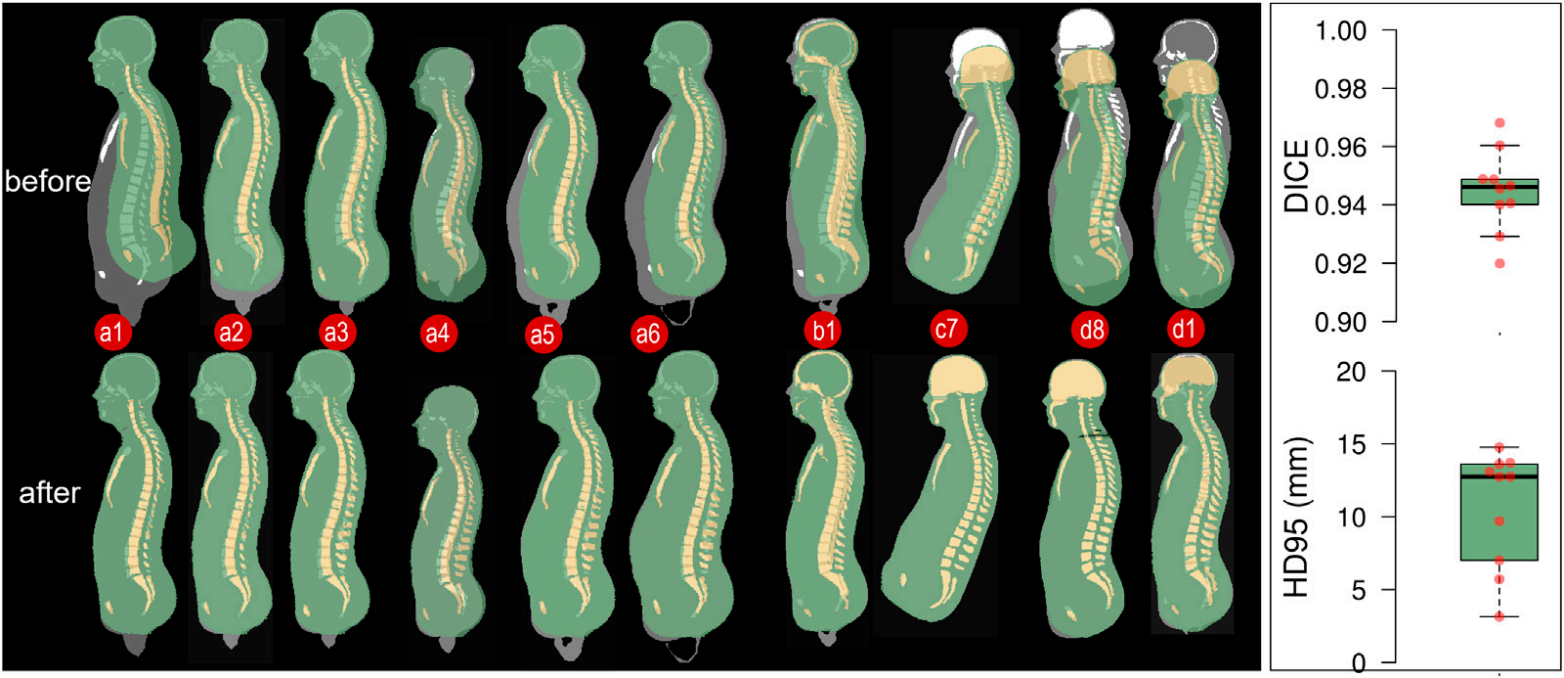
Overlay of voxelized images between baseline HBM and subjects (left upper row) with the gray image representing the subject (golden truth) and the colored image representing the baseline HBM. The same is shown after morphing where the colored images correspond to the personalized HBMs (left lower row). The right plot shows the DICE and HD95 values for the flesh part, with each dot representing one personalized model. It should be noted that the flesh part in this study refers to the total area minus the skeleton part, as indicated by the green image. The DICE values for the skeleton are not included since the values do not represent personalization accuracy due to topology differences between the baseline and HBM in the skeleton.

The ten personalized models are visualized in Figure 6. The voxelized images of the baseline HBM and subjects that were used in the registration process are shown in Figure 7. The images show a significant difference between the baseline HBM and subjects before morphing (Figure 7, left upper row), and after morphing, the difference is nearly imperceptible (Figure 7, left lower row), which indicates a high degree of personalization accuracy. This accuracy is further quantified with DICE and HD95 values (Figure 7, right plot). The mean DICE and HD95 for the ten personalized models are 0.94 and 10.63 mm, respectively.

### 3.2 Element quality

**FIGURE 8:**
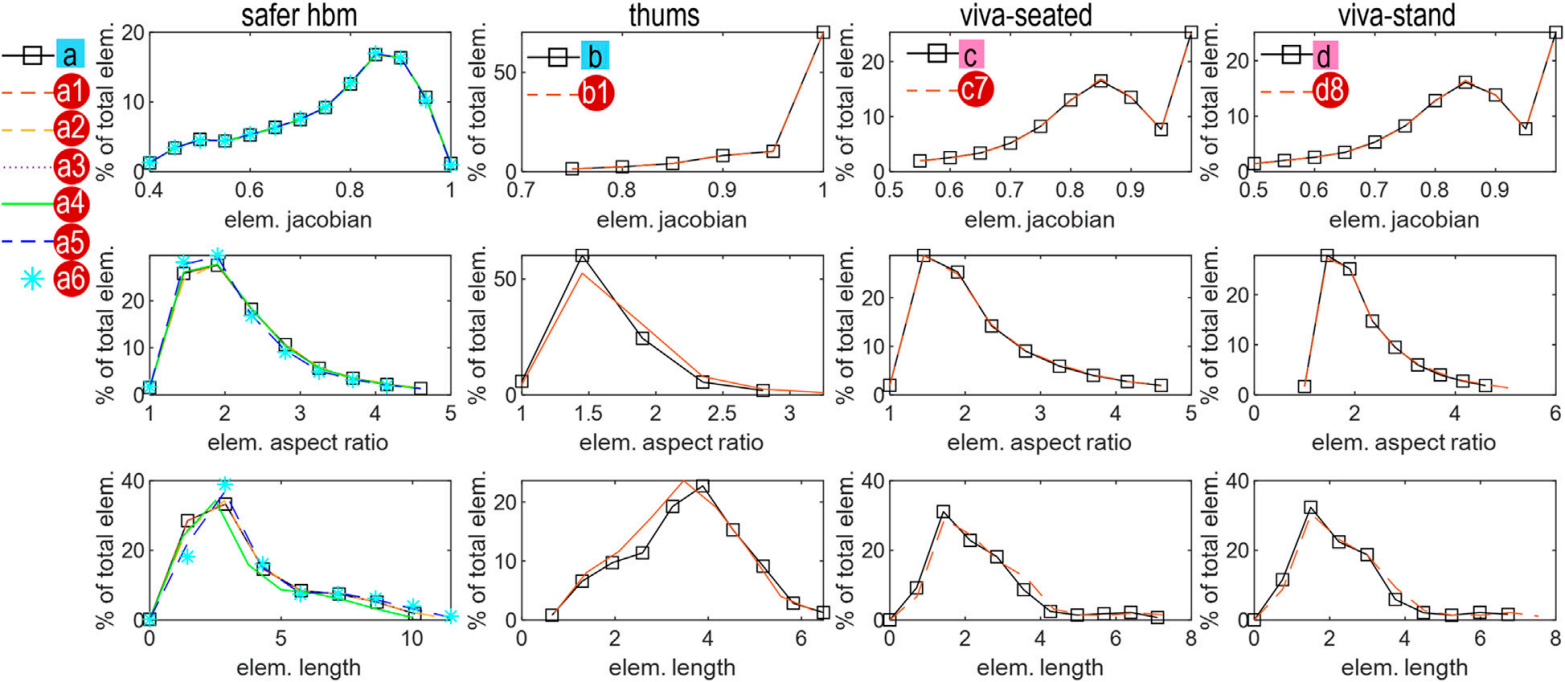
Histogram plot of the element quality of the ten personalized HBMs compared with its baseline, which includes Jacobian (first row), aspect ratio (second row), and element length (third row).

The ten personalized HBMs have comparable element quality with their respective baseline model in terms of Jacobian, aspect ratio, and element length (Figure 8).

### 3.3 Runnability test of personalized models

**FIGURE 9:**
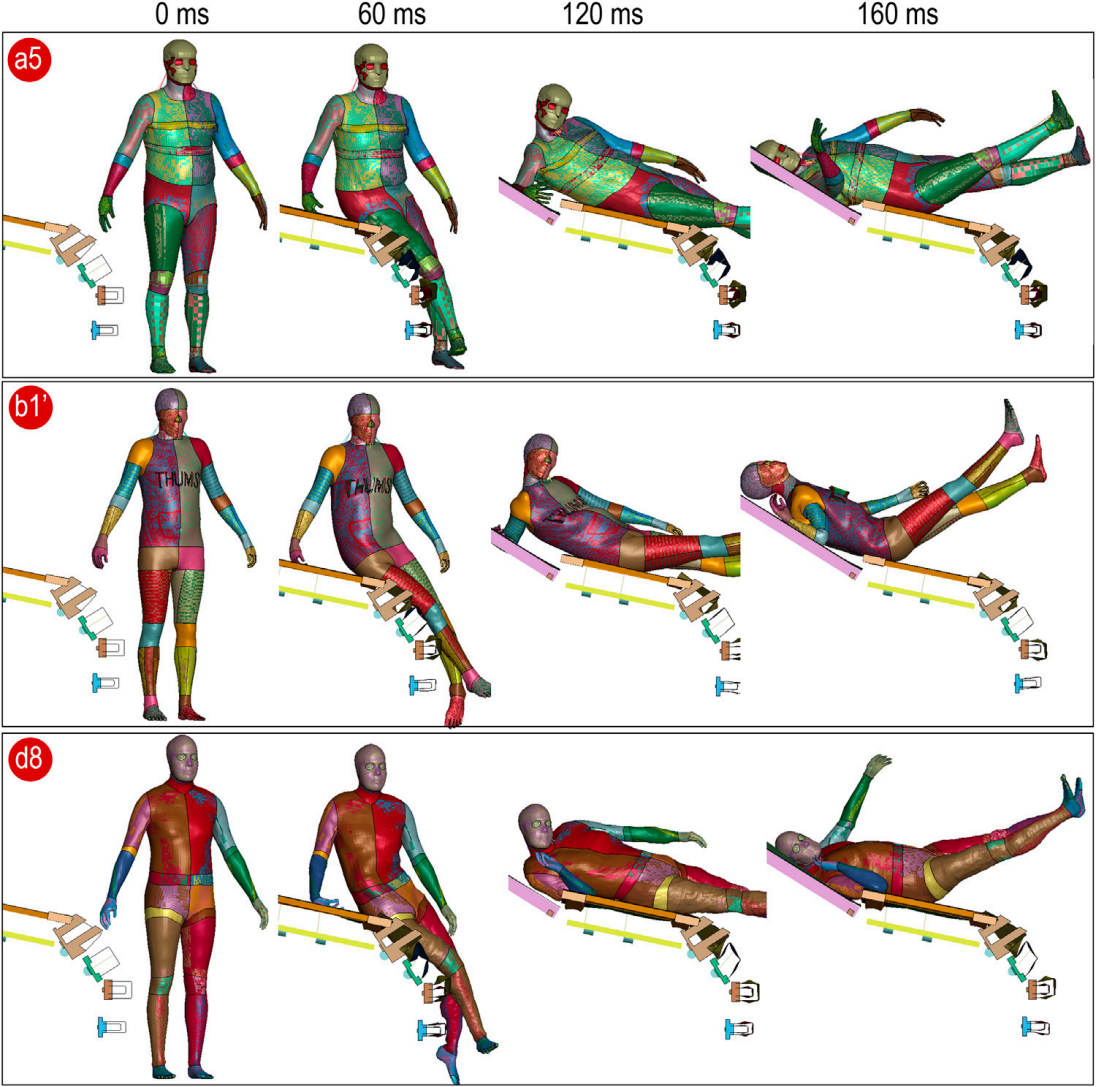
Runnability test of three personalized models simulating a side impact captured at 0, 60, 120, and 160 ms.

Three personalized HBMs (a6, b1', and d8) were selected for the runnability test, representing one baseline each from SAFER, THUMS, and VIVA+ in the standing posture. The models were subjected to a side impact using a generic vehicle buck model, showing that the personalized models are directly runnable (Figure 9) and can withstand such deformation with impact force comparable to a car accident.

### 3.4 Different HBMs personalized to the same 50th male

**FIGURE 10:**
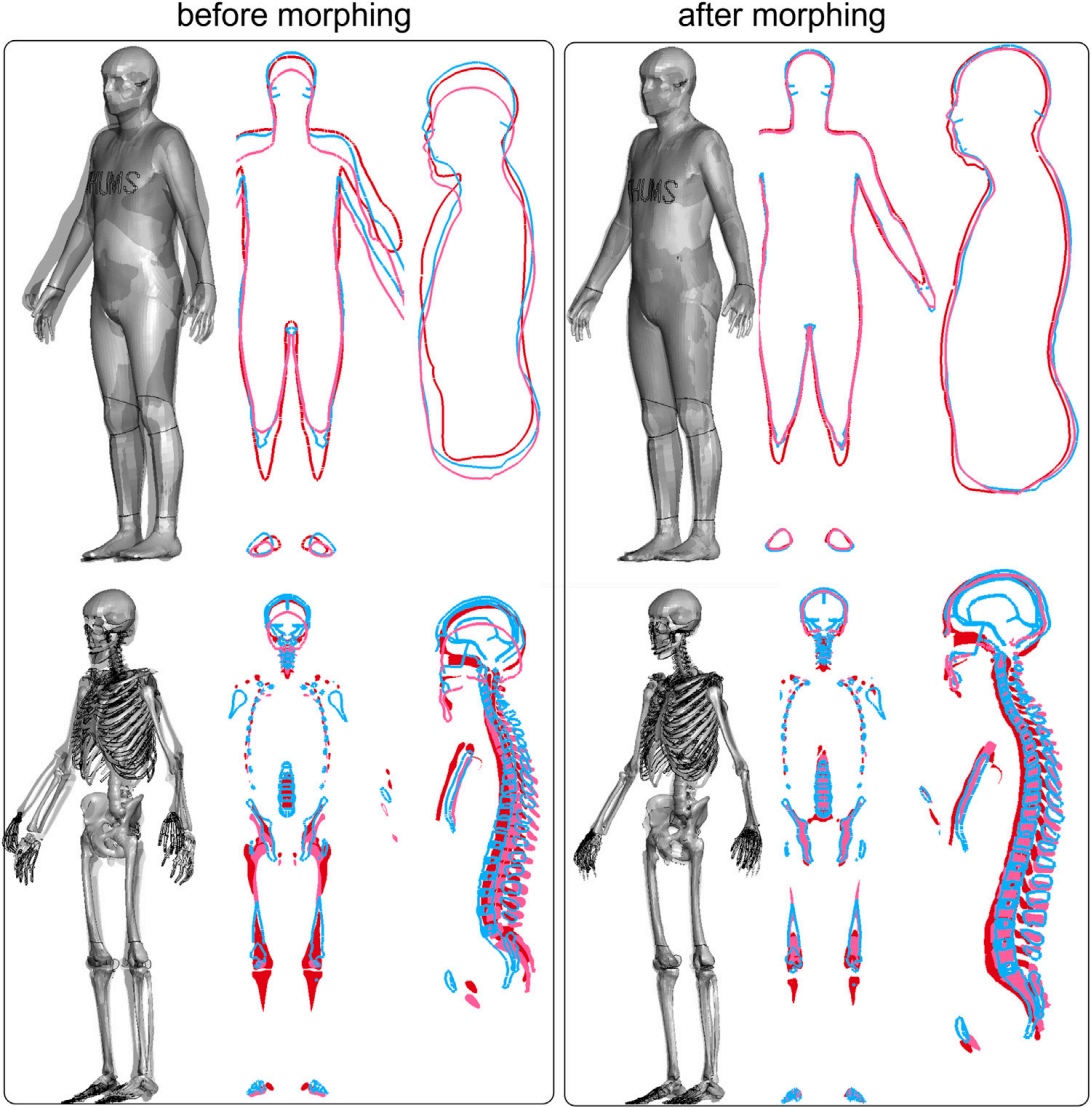
Overlay of three baseline HBMs before and after morphing to the same subject. The left box shows the three baseline HBMs before morphing (blue for SAFER, red for THUMS, and pink for VIVA+ standing). The right box shows the three same HBMs after morphing to *subj1*, which eliminates the geometrical differences among the HBMs.

The proposed method facilitates easy personalization of different HBMs into the same subject, thereby eliminating geometrical differences between the models. This is demonstrated by overlaying three personalized models (i.e., a1, b1, and d1) morphed to *subj1* from three baseline HBMs: SAFER HBM, THUMS, and VIVA+ female stand (Figure 10). The figures show observable geometrical differences among the HBMs before morphing (Figure 10, left box), while all models conformed to the same subject body shape and skeleton after morphing (Figure 10, right box).

### 3.5 Comparison to RBF morphing

**FIGURE 11:**
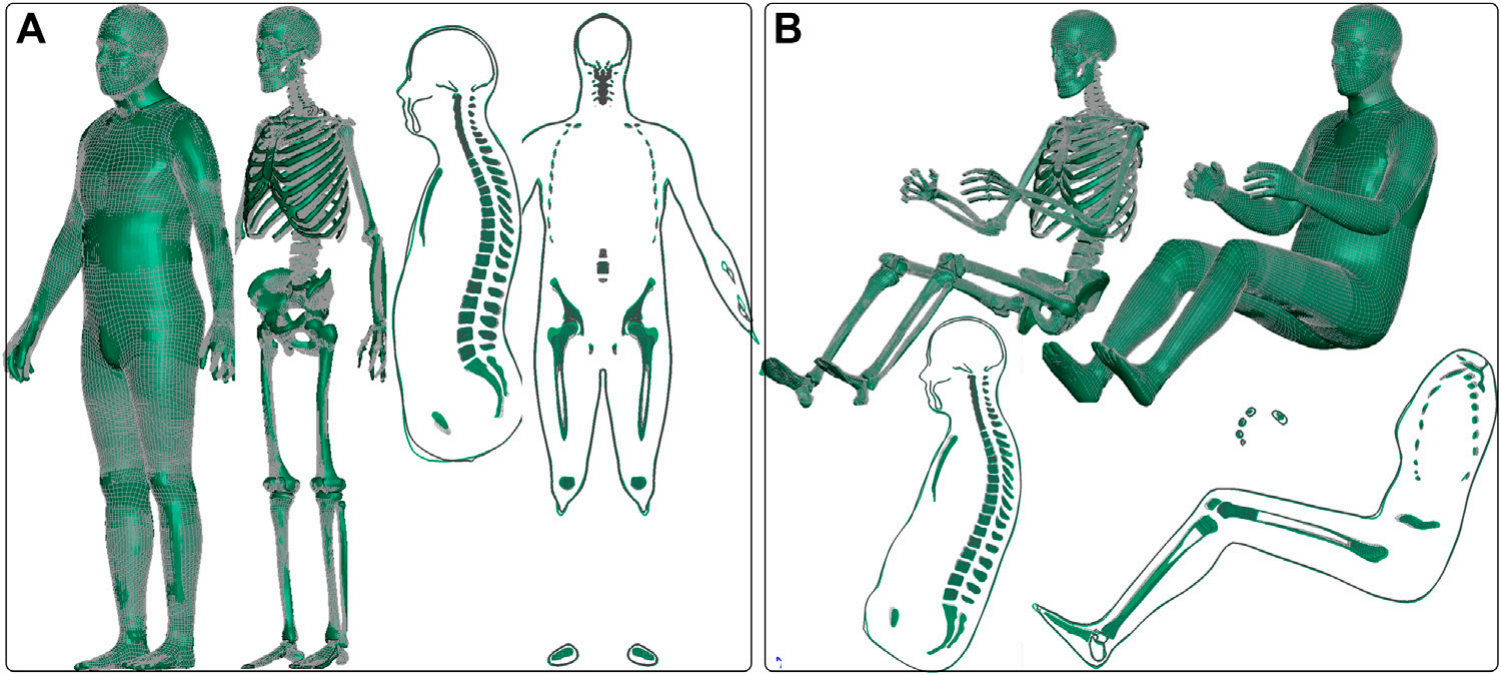
VIVA+ male 50th standing morphed from female via image registration in this study is visualized in gray wire compared with the morphed one using the RBF method by John et al. (2022), which is shown in green. **(A)** Similar comparison is made for the seated model **(B)**.

A comparison was made between two personalized models generated in this study (*c7* and *d8*) and models obtained with an RBF approach by John et al. (2022). The personalized models obtained using the current morphing method (represented by gray wire in Figure 11) are overlaid with the RBF models (represented by green in Figure 11), and the models were found to be very similar. The difference was quantified in Figures 8G, H (since subject 7 and 8 body shapes were reverse-engineered from the RBF models), with DICE values of 0.96 and 0.97, respectively, for the standing and seated models, and HD95 values of 5.72 mm for the standing model and 3.15 mm for the seated one.

## 4 Discussion

This study introduces an image registration–based method for personalization of HBMs and evaluates its performance in terms of personalization accuracy, element quality, and runnability. The ten personalized models achieved a high level of personalization accuracy with a mean DICE of 0.94 and a mean HD95 of 10.63 mm. The element quality of the personalized HBMs was comparable to their respective baseline, and the personalized models were directly runnable, without or only requiring minimal manual repair to avoid potential intersecting contacts in some morphed models. The method also enables easy personalization of different HBMs into the same subject, eliminating the geometric differences. A comparison with the RBF approach showed that the personalized models obtained using the current method are similar to those obtained using the RBF approach.

### 4.1 Fast and landmark-free HBM personalization method using image registration

The image registration–based method proposed in this study is landmark-free and distinguishes itself from traditional methods like RBF and kriging. Rather than relying on landmarks, this method converts surface models into binary images and employs Demons registration to obtain a displacement field that captures the anatomical difference between the subject and baseline HBM. This displacement field is then used to morph the baseline HBM to a target subject. One of the significant advantages of this method is that the resulting dense displacement field from registration captures detailed anatomy, and the Demons registration is “forgiving,” leading to valid element quality in personalized HBMs, even if there are holes or gaps in the voxelized binary image. By contrast, the RBF method often only uses thousands of landmarks since it involves the computationally heavy task of inverting a large matrix.

In image registration–based mesh morphing methods, there is a trade-off between personalization accuracy and element quality, determined by the chosen registration algorithm through the resulting displacement field. The Demons registration chosen in this study achieves a balance between personalization accuracy and element quality, yielding personalized HBMs with comparable element quality to the baseline. Of the steps, only the preprocessing module requires manual work and is quick to complete, while all other steps are automated and take minutes to complete. The extracted surface models for the baseline HBM can be reused when personalizing to other target subjects, making it easy to personalize baseline HBMs to new subjects.

### 4.2 Quality of personalized HBMs: element quality and contact

HBMs often contain many (up to thousands in some HBMs) surface contacts defined between body parts, e.g., the skeleton, inner organs, and flesh, to maintain separation. Intersecting contact surfaces will lead to locking contacts, which in turn will lead to unrealistic responses. Morphing HBMs can introduce new intersections, which must be carefully evaluated to ensure accurate results. The current morphing method generally introduces none or very few new intersections in the contact surface, depending on the quality of the baseline HBMs. For example, in the case of *d8* (morphing VIVA+ female to male), no new intersections were introduced, whereas for *b1* (THUMS morphing to *subj1*), two new contacting element intersections were introduced, and for the SAFER baseline to *subj1*, eight new contacting element intersections were introduced (illustrated in Supplementary Figure S2). These new intersections could nevertheless be manually repaired easily by translating the nodal coordinates of the intersecting elements to separate them.

### 4.3 Pipeline subtypes (Types I, II, and III)

The basic pipeline (Type I) was effective for nine out of ten evaluated cases, which included subjects with significant differences from the baseline, such as morphing a female baseline model to one of male (*c7* and *d8*) and high BMIs (*a6*). However, it failed when morphing SAFER HBM to *subj4*, resulting in a distorted foot. To solve this issue, Type II pipeline was used to first globally elongate the voxelized image of *subj4* before Demons registration. Type II pipeline can also involve multiple steps of Demons registrations for better accuracy in local regions of interest. Adding more regions, however, increases the risk of the decreased element quality. Type III pipeline is similar to Type I but has a “shielding” function to prevent certain regions, such as the head and foot, from being morphed.

### 4.4 Geometrical correction capacity facilitates extreme positioning and morphing of head models

Our method also provides easy geometric correction, as demonstrated by the successful correction of the THUMS chest (Figure 5) and SAFER buttocks (Supplementary Figure S1). It also facilitates extreme positioning of HBMs without the need for re-meshing. For example, the current morphing method when combined with the positioning tool Oasys PRIMER converted the SAFER HBM from a seated to standing version without re-meshing (Supplementary Figure S1). Converting a seated HBM to a standing one is challenging and often requires re-meshing or manual repair of the mesh (Peres, 2018; John et al., 2022). In a previous study, positioning of PIPER from seated to standing resulted in poor element quality at the joints, requiring re-meshing (Peres, 2018). A similar challenge was encountered when converting the VIVA+ female seated model to standing using an RBF-based approach, which resulted in problematic elements around the joints requiring manual repair (John et al., 2022).

Our approach for converting the SAFER occupant to a pedestrian (Lindgren et al., 2023) eliminates the need for manual repair. The process involves positioning the SAFER occupant to a pedestrian using the software Oasys PRIMER, similar to that done earlier for the PIPER HBM (Peres, 2018), but instead of re-meshing the flesh and skin, we used image registration to correct the shape and length. This produces a pedestrian model with a similar element quality as the baseline. Our method also allows for easy correction of models at the component level, as demonstrated by the successful correction of an 18YO PIPER head model. Our markerless method can be easily applied to personalize other existing HBMs, and it has a particular strength in generating subject-specific models when imaging data is available, such as full or partial body MRI images of the spine (Booth et al., 2022). The same pipeline used for the PIPER head (Supplementary Figure S5) can be used by segmenting CT/MRI images into binary images first.

### 4.5 Feasibility for morphing vehicle models

The proposed method is not limited to morphing HBMs and can be used to morph other types of models. To demonstrate this, we morphed an SUV into a sedan and *vice versa*, using voxelized images of the body and wheels while treating other parts, such as the windshield, as a single entity. The resulting morphed vehicle model is acceptable (Supplementary Figure S4B) and shows the potential of the method. However, the accuracy of the rear windshield of the morphed sedan is not ideal, as the SUV roof is morphed into the rear window. To improve accuracy, the windshield and body can be voxelized separately. It is also important to note that cars have different interiors and body parts, and our method is only suitable for rough estimation and morphing a baseline vehicle into a new car with similar structures.

### 4.6 Surface models of body shape and skeleton

We used a new open-source data set to obtain outer body shape models based on anthropometric data of height and weight. The skeletons were then automatically embedded using the open-source OSSO algorithm developed by researchers in the computer vision field (Keller et al., 2022). This approach offers an alternative to existing methods, such as the one used by John et al. (2022), where skeletons are embedded into outer body shapes based on bony landmarks using algorithms developed by Reed and Ebert. (2013).

We observed penetrations in the assembled skeleton and skin in some subjects, particularly in the pelvis area for subjects with lower BMI. However, the current method is forgiving, as it did not cause element quality problems in all the evaluated cases. In fact, including the skeleton would more likely lead to poor element quality than skin-only. As an alternative, skin-only body shapes could be used during morphing if relevant. Our results show that morphing using skin-only body shapes leads to acceptable personalized models. In this case, HumanShape.org is a valuable database for obtaining skin-only body shapes. However, including a skeleton would allow for more accurate personalization, especially for subjects with higher BMIs.

### 4.7 Limitations and future work

The current method is efficient for personalization and can position certain body parts, such as the arms and trunk. However, its positioning capacity is limited, particularly in regions where the source and target images do not overlap, such as in the hands. As a result, subjects’ arms must be positioned similar to baseline HBMs before morphing. The accuracy of personalization depends on the image registration algorithm used. The currently used Demons registration achieves good overall accuracy in the personalized models. However, some areas, such as the skeleton, may not be as accurate, where the rib cage deviates from the subject. This is potentially problematic if conducting a study on rib fractures, as previous research has shown that rib cage shape has a significant impact on fracture risk (Larsson et al., 2022). To address this issue, local morphing of specific regions can be performed to improve accuracy. Finally, the currently chosen Demons registration leads to a balanced personalization accuracy and element quality, but alternative algorithms, such as DRAMMS (Ou et al., 2011), could be explored in future studies.

## 5 Conclusion

In conclusion, this study introduces a new and efficient landmark-free method for personalizing HBMs using image registration. This automated approach allows for rapid personalization of HBMs to new subjects, incorporating the body shape and skeletons. The resulting personalized HBMs have element quality comparable to the baseline. The method also has strength in geometrical correction and facilitates extreme positioning of HBMs when combined with other positioning software. The proposed method has been demonstrated to be an efficient and robust approach for personalizing a range of HBMs, with potential applications beyond this. It also has particular strengths in geometric correction.

## Data Availability

The raw data supporting the conclusion of this article will be made available by the authors, without undue reservation.
