# Supplementary material for: Personalization of human body models and beyond via image registration
Source: Front Bioeng Biotechnol. 2023 May 19;11:1169365. doi: 10.3389/fbioe.2023.1169365 (PMC10236199; doi:10.3389/fbioe.2023.1169365)
Supplement: Supplementary file 1 [file DataSheet1.docx]

Supplementary Material


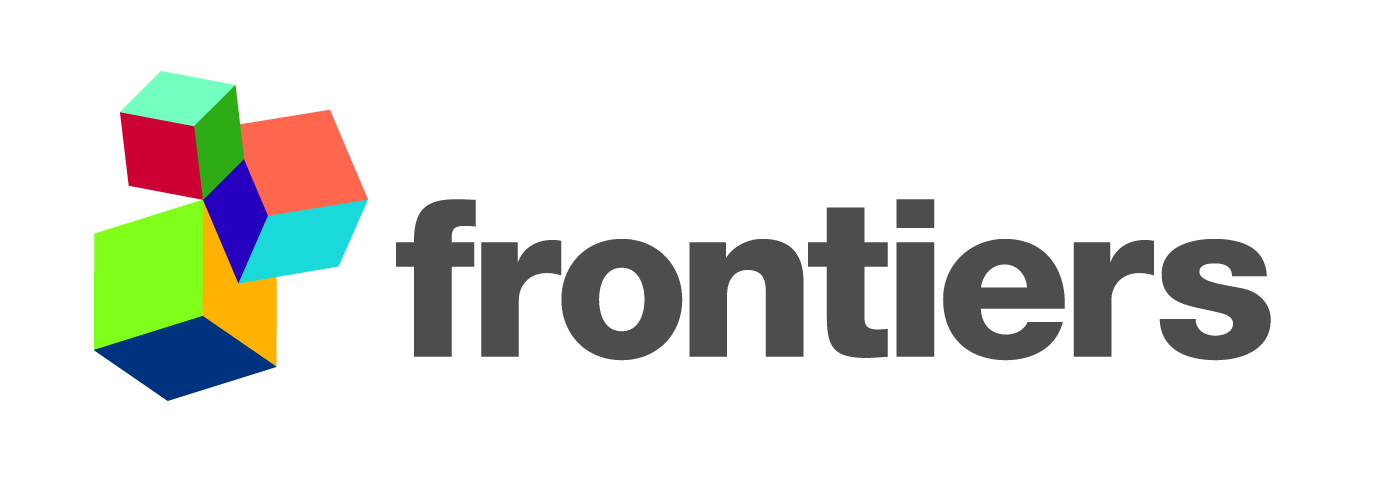


# Personalization of human body models and beyond via image registration

**Xiaogai Li**^1^***, Qiantailang Yuan**^1†^**, Natalia Lindgren**^1†^**, Qi Huang**^1^**, Madelen Fahlstedt**^2^**, Jonas Östh**^3,4^**, Bengt Pipkorn**^5,4^**, Lotta Jakobsson**^3,4^**, Svein Kleiven**^1^

^1^Division of Neuronic Engineering, Department of Biomedical Engineering and Health Systems, KTH Royal Institute of Technology, Huddinge 141 52, Sweden

^2^Mips AB, Täby, Sweden

^3^Volvo Cars Safety Centre, Gothenburg, Sweden

^4^Division of Vehicle Safety, Department of Mechanics and Maritime Sciences, Chalmers University of Technology

^5^Autoliv Research, Vargarda, Sweden

*Corresponding address xiaogai@kth.se

^†^These authors contributed equally to this work

Supplementary Material Summary

**Appendix S1: SAFER HBM morphing**

**Appendix S2: Morphing vehicle to demonstrate the application of the method beyond HBMs**

**Appendix S3: Morphing PIPER head to demonstrate geometrical correction capacity**

## Appendix S1: SAFER HBM morphing

Further steps for SAFER HBM morphing are shown here since it further illustrates the use of a Type III shielding pipeline and illustrates the geometrical correction of this method.

### *Baseline 50^th^ pedestrian SAFER HBM*

Type III “shielding” pipeline and geometrical correction demonstration. Detailed steps are presented earlier (Lindgren et al. 2023).


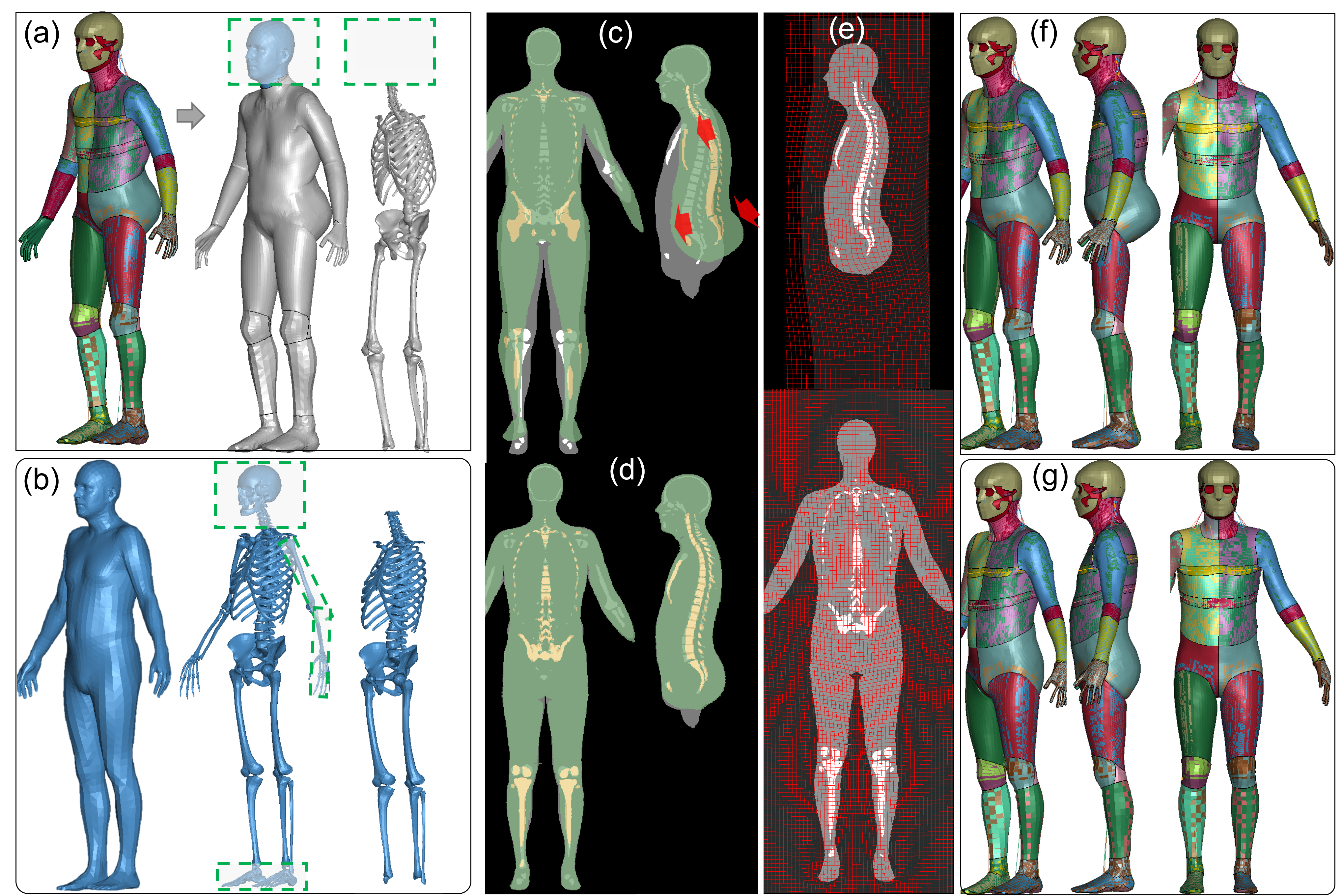


**Figure S1** Type III pipeline with “shielding” for the generation of pedestrian SAFER HBM. SAFER HBM occupant posture is positioned to pedestrian using Oasys PRIMER led to reasonable element quality of the flesh but caused distorted buttocks and a shorter total length (a). This model was then morphed to subj1 body shape (b) with “shielding” of the head and foot. The baseline (color) and subject image (gray) are overlayed before (c) and after the registration (d). The displacement field obtained from the registration (e) is used to morph the PRIMER-positioned model (f) to a final pedestrian version of the SAFER HBM (g).


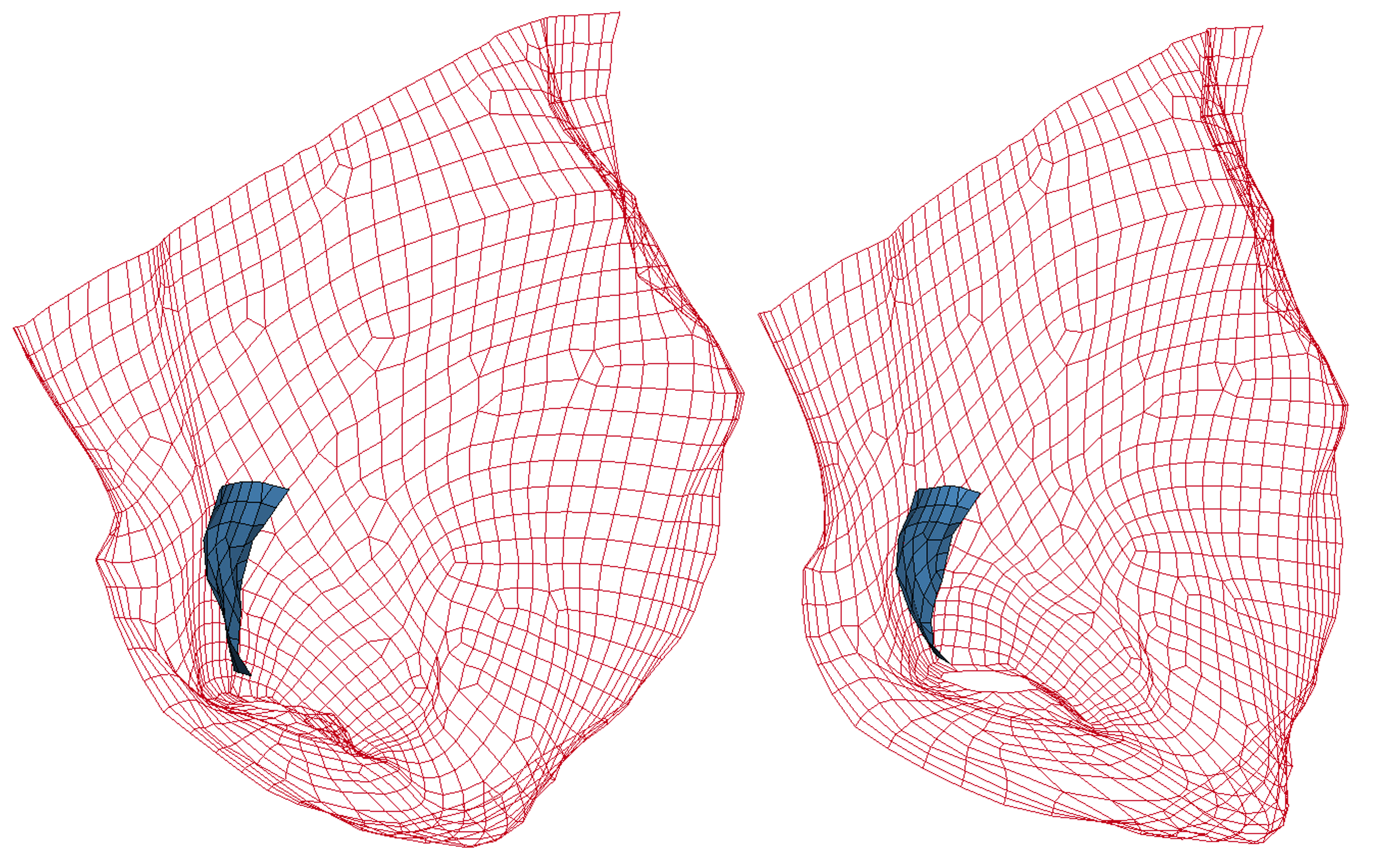


**Figure S2** Illustration of new intersection contacts introduced when morphed from (f) to (g) in above Figure S1 with eight new intersections introduced between the pelvis soft tissue (red) and superior iliofemoral ligament (blue) that was introduced by the morphing.

### *Baseline SAFER personalized to subjects 2-6*

For subjects 2 to 6, the same procedure could have been done as for the baseline model as described above. However, we used the baseline SMPL subj1 body surface as a “bridging” to personalize to other subjects to avoid modifying meshes for shielding. For example, for morphing the SAFER HBM to subject 6, instead of using the surface model extracted from SAFER HBM as shown in **Figure S1**, an intermediate surface model (**Figure S3**b) is used to utilize since it has same topology with other subjects’ surfaces (**Figure S3**c) and voxelized images (**Figure S3**d) which is used for generating the personalized models for subjects 2 to 6.


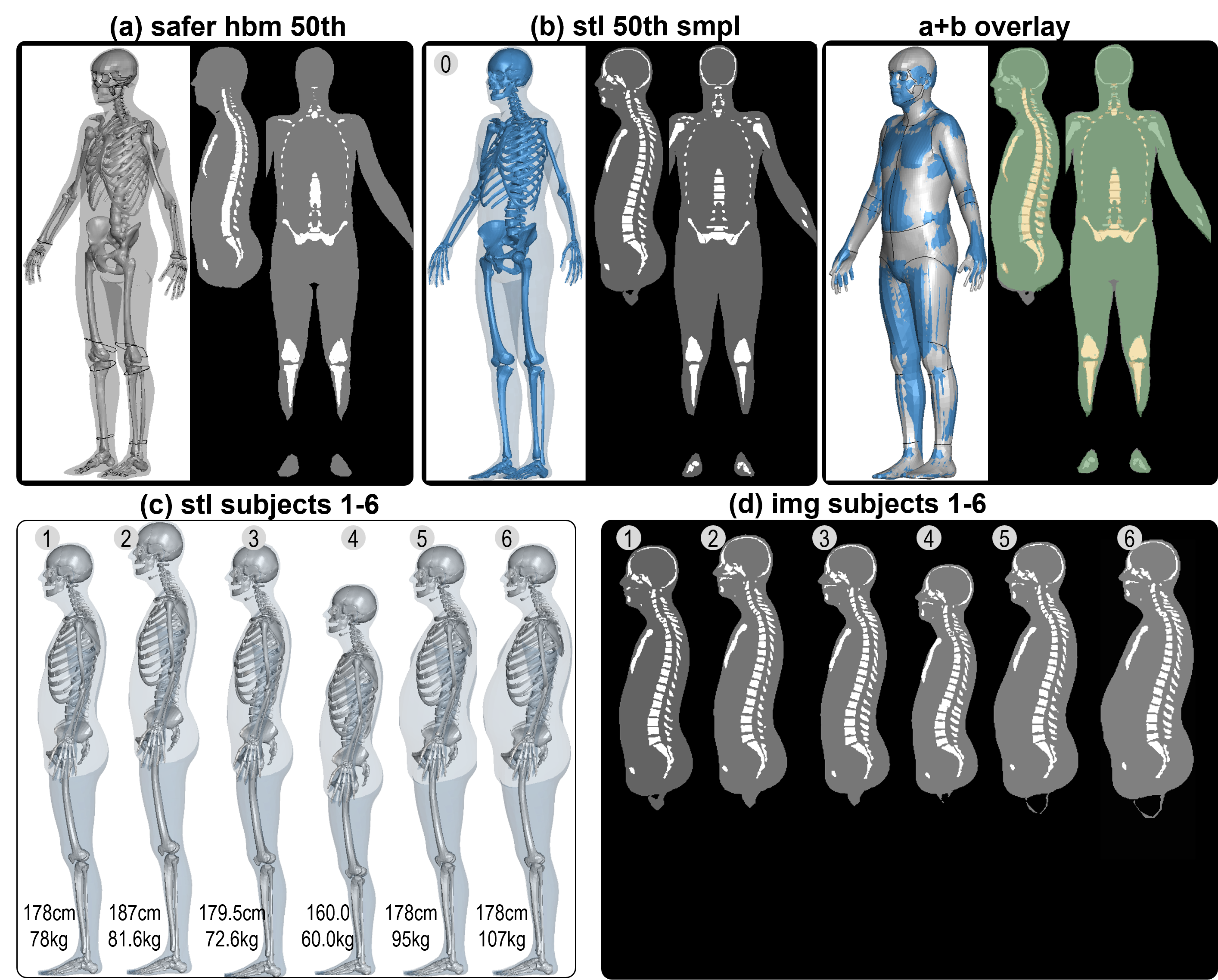


**Figure S3** Baseline SAFER HBM (a) personalized to subjects (c) using “intermediate” body surface (b) to bridge as it corresponds to the baseline HBM.

## Appendix S2: Morphing vehicle to demonstrate the application of the method beyond HBMs

Vehicle models are needed to pair with HBMs to study human injuries during traffic accidents. Based on the typical characteristics of considerable cases recorded in the GDV (German Insurance Association–Institute of Vehicle Safety) database, pedestrian head injuries are the most common severe and fatal injuries in car-to-pedestrian accidents (Kramlich et al. 2002). The front-end structure of vehicles including the car hood and windshield has been identified as one of the main contact sources for pedestrian head injuries (Huang et al. 2010). Various vehicle front-end structures can both influence the head kinematics and impact location, which further affect head injuries. Thus, case-specific vehicle models are needed for more reliable predictions of pedestrian head impact conditions. Some simplified vehicle models including BUCK (Pipkorn et al. 2014), generic vehicle (Klug et al. 2017) FE models, and parametrized multi-body model (Liu et al. 2022) have been developed and widely used in car-to-pedestrian simulations. Compared with the above-simplified models, detailed vehicle models are more accurate and reliable in accident reconstructions considering the non-ideal head impact conditions in real accidents. However, developing a detailed vehicle model from scratch is similarly challenging as HBMs.

To highlight the application of the current method and the importance of vehicle models in traffic safety, we show the geometry of an SUV and sedan that can be morphed between each other. First, two baseline vehicle FE models including the 2020 Nissan Rogue (SUV) and 2015 Toyota Camry (sedan) (**Figure S4**a) were downloaded from an open-source dataset (https://www.ccsa.gmu.edu/models/) serving as a baseline model to be morphed. Second, the surface models of the two vehicles were extracted which were then voxelized into binary images representing the outer shape. Third, a Type I pipeline Demons registration was performed from which a displacement field is obtained, which was then used to morph a baseline mesh. When morphing the SUV into a sedan, the SUV image serves as *fixed* image, and sedan image as a *moving* image, and vice versa when morphing the sedan to SUV.

The baseline model SUV (2020 Nissan Rougue) (**Figure S4**a left) was morphed into a Toyota sedan with the same topology just the shape changed (**Figure S4**b left). Vice versa, the baseline sedan (2015 Toyota Camry) (**Figure S4**a right) was morphed into an SUV (**Figure S4**b right). Voxelized binary images of the two vehicles are overlayed (**Figure S4**c) showing the difference in geometry between the two vehicles before morphing. Further details of the displacement field and warped image when morphing the SUV to the sedan is provided here. The obtained displacement field (**Figure S4**d) is used to displace the nodes of the SUV to that of the sedan). After morphing the two images overlay shows an almost invisible difference indicating the personalization accuracy (**Figure S4**e).


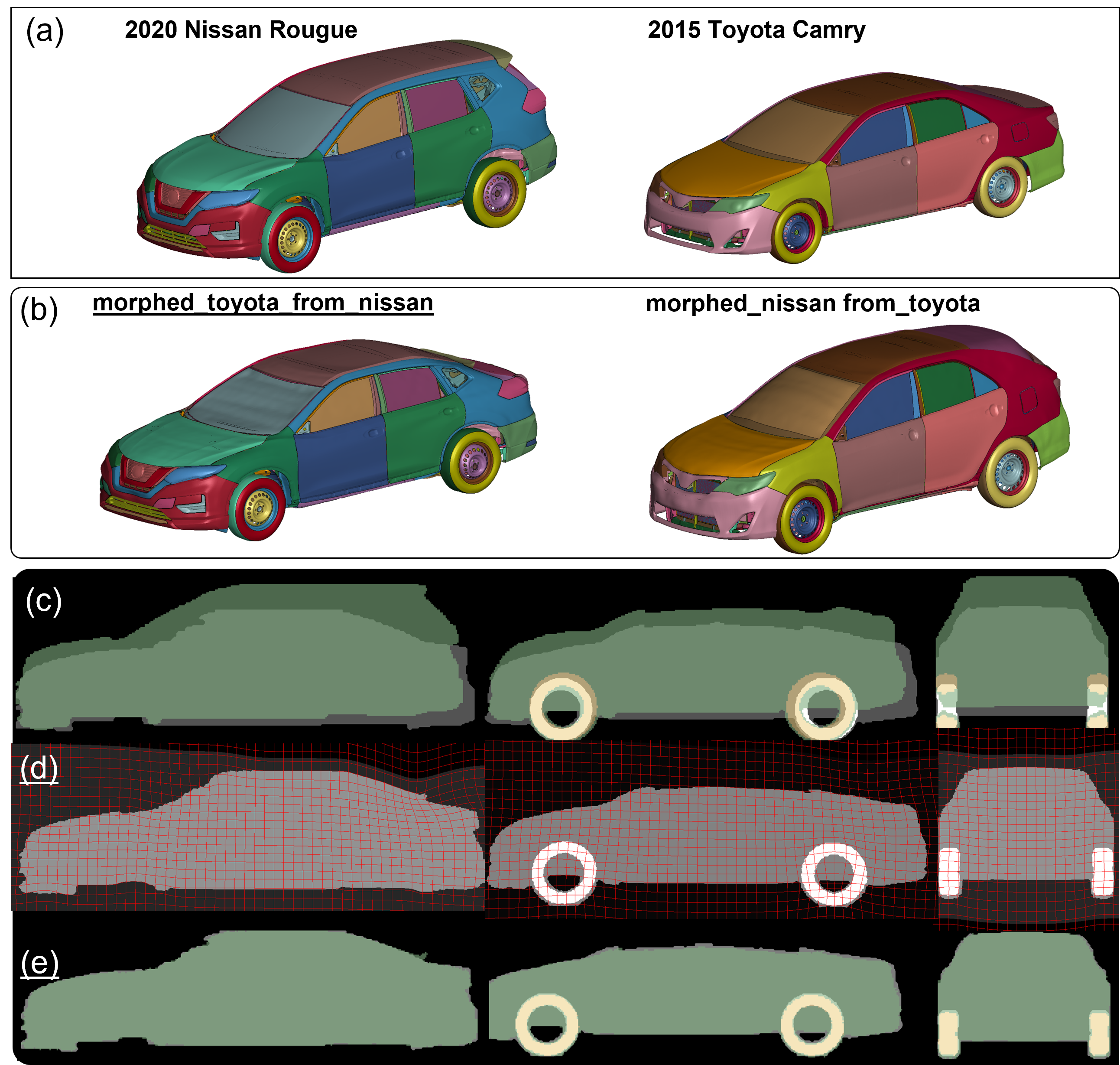


**Figure S4** (a) Baseline vehicle models 2020 Nissan Rougue (left) and 2015 Toyota Camry (right); (b) morphed Toyota model from Nissan (left) and morphed Nissan model from Toyota (right) using Type I pipeline; (c) voxelized images of the Nissan (in color) and Toyota (in gray) before image registration showing the differences between the two vehicle models; (d) obtained displacement field from Demons registration that is used to morph (displace the nodes) the Nissan SUV to Toyota Sedan as shown at the rear end pushed down; (e) voxelized image of the Toyota Sedan overlay with warped Nissan to Toyota (corresponding to the personalized vehicle model) showing almost overlayed perfectly indicate its accuracy.

## Appendix S3: Morphing PIPER head to demonstrate geometrical correction capacity

The PIPER software allows morphing a 6yo PIPER child model from 1.5 to 12 years old using nonlinear kriging based on statistical data. But the software offers the possibility to go up to 18 years old. However, above 12yo is based on linear interpolation and the 18yo head has flatted occipital part (**Figure S5**a). For this, the baseline model is the directly morphed 18yo from the PIPER tool with a flat head. The subject's MRI is downloaded from an open-source dataset (Evans et al. 2006). Here we show the geometry can be corrected with this current method. First, the surface model of the flat head is extracted and voxelized into a binary image. Second, the MRI of the 18yo subject is also segmented for the cranial image. Third, Type I pipeline Demons registration is performed with baseline as a *fixed* image and subject cranial image as a *moving* image, from which a transformation is obtained, which is then used to morph the baseline mesh resulting in an upgraded mesh.

The 18yo head model directly morphed from the PIPER software is shown in **Figure S5**a with a flat head at the back, which is corrected by morphing to a subject of 18yo (**Figure S5**b) by mesh morphing. The cranial of the subject is segmented (**Figure S5**c) overlaying with the cranial of the baseline head showing the difference before morphing (**Figure S5**b). The obtained displacement field (**Figure S5**d) defined on the space of the baseline mesh is used to displace the nodes of baseline to the subject (**Figure S5**e) showing almost invisible difference indicating its personalization accuracy with a DICE of 0.97, and HD95=2.53mm.

*
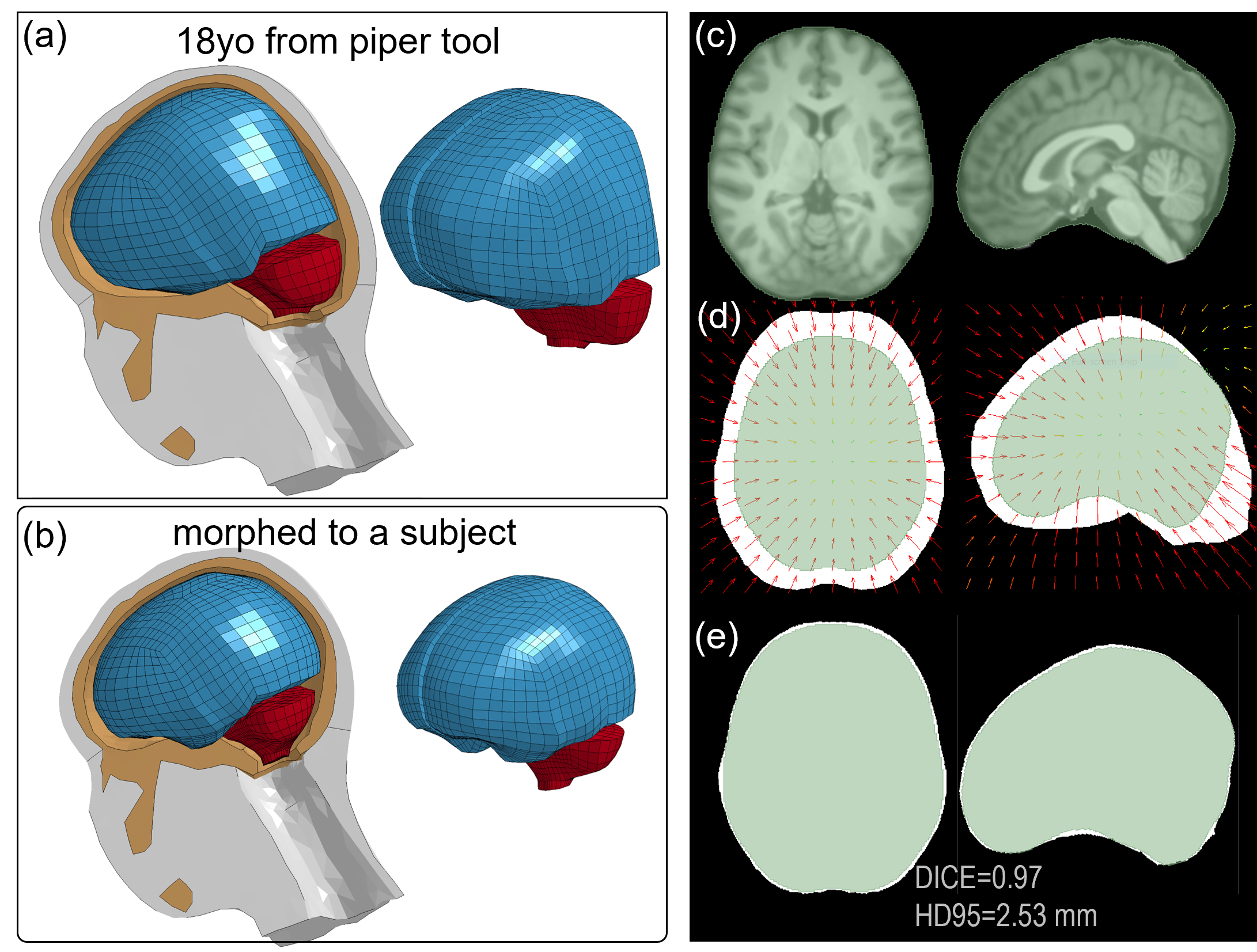
*

**Figure S5** (a) PIPER head model of 18yo from PIPER software with a flatted head; (b) morphed to a subject head according to MRI image; (c) MRI of the adult subject with a cranial segmented overlay of the original image; (d) the subject cranial (green) overlay with the voxelized image of the 18yo PIPER (white color) showing the difference before morphing and the arrows indicate the displacement field obtained from Demons registration that morphed the head to the subject correcting the flatted occipital part; (e) after morphing the morphed head model voxelized image is overlaid with the subject indicating its personalization accuracy.
